# Deep learning analysis of urine-derived stem cell mitochondrial morphology as a non-invasive Alzheimer’s disease biomarker

**DOI:** 10.1016/j.neurot.2025.e00813

**Published:** 2025-12-16

**Authors:** Ran Yan, Wenhua Zhang, Wenjing Wang, Jiaqi Wu, Jun Zhang, Yingjie Xu, Wei Xu, Wen Yang

**Affiliations:** a Department of Neurology and Institute of Neurology, Ruijin Hospital, Shanghai Jiao Tong University School of Medicine, Shanghai, China; b Department of Biochemistry and Molecular Cell Biology, Shanghai Key Laboratory for Tumor Microenvironment and Inflammation, Shanghai Jiao Tong University School of Medicine, Shanghai, China; c RNAcure Biopharma, Shanghai, China; d Department of Biology, Bates College, Lewiston, ME, USA; e School of Future Techonology, Shanghai University, Shanghai, China; f Tencent AI Lab, Shenzhen, 518057, China

**Keywords:** Alzheimer’s disease, Mitochondrial morphology, Artificial intelligence, Urine-derived stem cell

## Abstract

Alzheimer’s disease (AD), closely associated with mitochondrial dysfunction, currently lacks convenient and non-invasive biomarkers for mitochondrial assessment. In this study, we developed an artificial intelligence framework leveraging live urine-derived stem cell (USC) mitochondrial fluorescence imaging to investigate differences between cognitively impaired individuals (AD and mild cognitive impairment (MCI)) and cognitively normal (CN) subjects. Mitochondrial fluorescence images from living HeLa cells were first segmented, and two binary classification models based on the ResNet-18 convolutional neural network were trained to identify mitochondrial hyperfission and hyperfusion relative to normal morphology. The models demonstrated robust performance in detecting intermediate mitochondrial states during validation. When applied to USCs, the system effectively distinguished mitochondrial patterns associated with cognitive impairment, highlighting its potential for the early detection of Alzheimer’s disease and merits further validation in larger, independent cohorts.

## Introduction

Alzheimer’s disease (AD) is an age-related progressive neurodegenerative disorder primarily characterized by cognitive decline, including impairments in memory, thinking, and reasoning. It represents the most common form of dementia. Despite extensive research, the detailed pathogenesis of AD remains unclear. The leading hypotheses include the amyloid-beta (Aβ) cascade and tau hypotheses, however, pharmacological interventions targeting these pathways have produced limited success [[1], [2], [3]]. Increasing evidence suggests that mitochondrial dysfunction contributes to AD pathogenesis [4,5]. Positron emission tomography (PET)-CT studies have revealed abnormalities in mitochondrial complex I activity in the brains of AD and mild cognitive impairment (MCI) patients [[6], [7], [8]], establishing a clear association between mitochondrial dysfunction and AD [5,9].

Mitochondrial dysfunction, a well-established cornerstone of Alzheimer’s disease (AD) pathology, is increasingly recognized as a systemic alteration that occurs not only in the brain but also in peripheral systems. This view is supported by the geroscience perspective, which posits that shared hallmarks of aging, like mitochondrial decline, underpin multiple age-related diseases [10,11]. Indeed, patients with AD and MCI show reduced expression of mitochondrial-related genes in peripheral blood [12], accompanied by altered mitochondrial function [13]. Moreover, agents targeting oxidative stress, inflammation, and mitochondrial dysfunction have shown potential neuroprotective effects in neurodegenerative disorders [14,15].

However, current assessments of mitochondrial changes in AD mainly rely on PET imaging [6,7,16] or blood-based biomarkers [12,13], which are often costly, invasive, or limited to single time points. Therefore, there is a pressing need for non-invasive, accessible, and dynamic approaches to evaluate mitochondrial health. Human-derived cells are widely used in AD research [17,18]. Notably, urine-derived stem cells (USCs) offer a unique advantage: they provide living, metabolically active cells that can be non-invasively obtained and cultured as urine is a traditional source for biomarkers [[19], [20], [21], [22], [23], [24], [25], [26]]. This enables direct functional assessment of mitochondrial networks, positioning USCs not only as a biomarker source but also as a dynamic, patient-specific model system for studying systemic mitochondrial pathophysiology in AD.

Mitochondria continuously undergo fusion and fission to maintain cellular homeostasis [27], and their function is closely linked to morphological dynamics [28]. During these processes, mitochondrial morphology shifts among diverse forms, including spheroidal, rod-shaped, twisted, and branched structures [[29], [30], [31]]. Under conditions such as oxidative stress or apoptosis, mitochondrial morphology can undergo profound changes [32,33]. Structural and functional mitochondrial abnormalities are widely observed in AD [[34], [35], [36], [37]], Parkinson’s disease [38] and aging [39]. Therefore, directly examining mitochondrial morphology in cells derived from AD patients may provide valuable evidence to further elucidate the mitochondrial cascade hypothesis and aid early AD diagnosis.

Traditional analyses of mitochondrial morphology largely rely on qualitative assessments or basic quantitative measures [40]. For example, length-based scoring systems have been used to describe the fission-fusion balance, but these approaches remain simplistic and fail to capture the multidimensional nature of mitochondrial dynamics [41]. Recent advances in imaging and computational techniques have enabled more sophisticated morphological analyses. New software and algorithms can objectively segment individual mitochondria, classify their morphology, and quantify parameters such as count, length, and width [41,42]. These tools can identify condition-specific morphological differences at both mitochondrial and cellular levels [[41], [42], [43], [44]]. However, most existing methods focus on isolated mitochondria, neglecting the integrated network behavior that underlies mitochondrial function within cells.

**Fig. 1:**
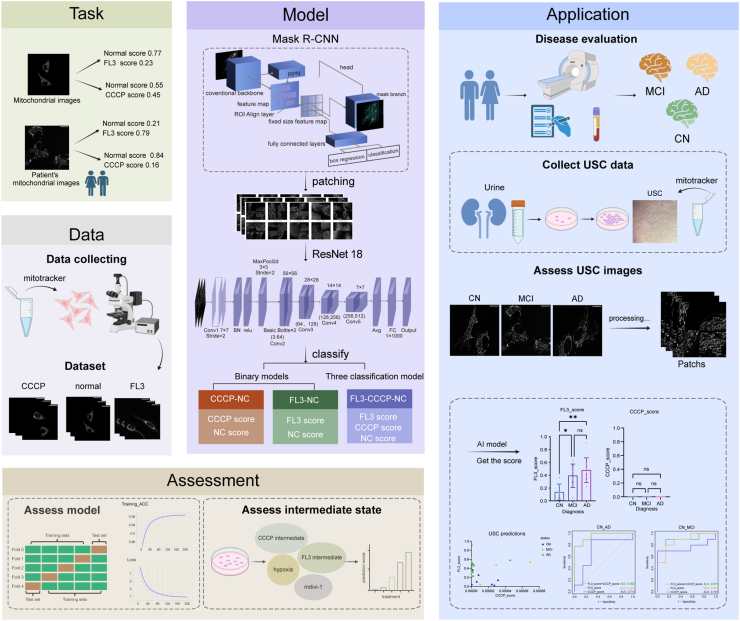
The overall design of the research.

This study aims to develop artificial intelligence (AI) models capable of accurately and unbiasedly identifying mitochondrial morphology in USCs from AD patients, thereby advancing understanding of the relationship between AD and mitochondrial homeostasis. The overall workflow of our methodology is illustrated in Fig. 1.

## Methods and Materials

### HeLa cell culture

HeLa cells were cultured in Dulbecco’s Modified Eagle Medium (DMEM, L110KJ, Basalmedia) supplemented with 10 ​% Fetal Bovine Serum Gold (FBS; 40130ES761, Yeasen Biotechnology) and 1 ​% Penicillin-Streptomycin Solution (S110JVIC, Basalmedia). For imaging, a colorless DMEM medium (L140KJ, Basalmedia) was used with the same supplementation. Cells were maintained in a humidified incubator (HF90, Heal Force) at 37 ​°C with 5 ​% CO_2_.

### Preparation for HeLa cell mitochondrial imaging

HeLa cells were seeded at a density of 2–4 ​× ​10^4^ ​cells/ml, with 400 ​μl of cell suspension plated onto glass-bottom dishes (J40204, BQ-LAB). After 24 ​h of incubation, the culture medium was replaced with fresh DMEM containing 50 ​nM MitoTracker® Deep Red FM (M22426, Invitrogen) for mitochondrial staining. Cells were incubated with the dye for 30 ​min, followed by replacement with colorless DMEM complete medium for recovery for at least 2 ​h before imaging.

### Drug treatment

For pharmacological perturbations, HeLa cells were treated with carbonyl cyanide *m*-chlorophenylhydrazone (CCCP; B5003, APEXBIO) for 2 ​h, or exposed to hypoxic conditions for 3 ​h after mitochondrial staining. Treatments with FL3 (provided by Laurent Désaubry’s laboratory) and mdivi-1 (M0199, Sigma) were performed for 24 ​h and 12 ​h, respectively, prior to mitochondrial staining. Colorless DMEM medium was used during imaging to minimize background fluorescence.

### Participants

Participants were recruited from the Neurology Clinic of Ruijin Hospital, affiliated with Shanghai Jiao Tong University School of Medicine. The study was approved by the Ruijin Hospital Ethics Committee (Approval No. 2018-204). All participants provided written informed consent prior to enrollment. Cognitive function was independently assessed by at least two experienced neurologists using a standardized battery of neuropsychological tests, including the Mini-Mental State Examination (MMSE) and Montreal Cognitive Assessment (MoCA).

#### Inclusion criteria

Eligible participants were aged over 50 years and provided informed consent. Individuals were classified into cognitively normal (CN), mild cognitive impairment (MCI), or Alzheimer’s disease (AD) groups. The clinical definitions of CN and MCI followed the syndromic stages outlined in the 2018 National Institute on Aging and Alzheimer’s Association (NIA-AA) research framework [45], while the diagnosis of AD was based on the 2024 NIA-AA diagnostic criteria [46].

#### Exclusion criteria

Participants were excluded if they had any condition that could interfere with cognitive evaluation, including metabolic or infectious encephalopathies, major cerebrovascular disease, other neurodegenerative disorders (e.g., Parkinson’s disease, multiple system atrophy with dementia), psychiatric disorders, congenital intellectual disability, severe systemic organ dysfunction, or a history of substance or alcohol dependence.

#### Cohort characteristics

A total of 26 participants were enrolled, comprising 7 CN, 10 MCI, and 9 AD individuals. Demographic and clinical data are summarized in Table 1. To reduce inter-individual variability, strict inclusion and exclusion criteria were applied. Baseline characteristics were analyzed using one-way ANOVA and the Kruskal-Wallis test. No significant group differences were found in age or sex distribution (*P* ​> ​0.05), whereas MMSE scores showed a progressive decline from CN to MCI and AD (*P* ​< ​0.0001).

**Table 1:** Patients’ information.

Characteristics	CN	MCI	AD	P value
Number of values	7	10	9	
Age (years, mean ​± ​SD)	63.57 ​± ​10.01	72.2 ​± ​4.71	72 ​± ​7.78	>0.05
Male (number/ratio)	1 (14.3 ​%)	3 (30.0 ​%)	4 (44.4 ​%)	>0.05
Female (number/ratio)	6 (85.7 ​%)	7 (70.0 ​%)	5 (55.6 ​%)	
MMSE (mean ​± ​SD)	29.43 ​± ​0.53	27.4 ​± ​1.51	18.56 ​± ​4.98	<0.0001

Abbreviations: MMSE, Mini-mental State Examination; SD, standard deviation.

### Culture of urine-derived stem cells

Urine-derived stem cells (USCs) were chosen as the biological model in this study for their combined biological relevance and clinical practicality. As metabolically active progenitor cells, USCs offer a robust system for assessing mitochondrial physiology. In addition, their non-invasive acquisition makes them well suited for scalable, patient-friendly applications such as longitudinal monitoring and early disease detection.

Midstream urine samples were collected from participants. Each sample was supplemented with 5 ​% Penicillin-Streptomycin solution and centrifuged at 400 ​g for 10 ​min. The cell pellet was resuspended in phosphate-buffered saline (PBS), centrifuged again under the same conditions, and the final pellet was resuspended in REGM/DMEM medium. The suspension was then transferred to a 0.1 ​% gelatin-coated culture dish (V900863, Sigma) and left undisturbed for 5 days to allow primary cell attachment and colony formation. Once colonies appeared, cultures were maintained with half-medium changes.

REGM complete medium: 50 ​mL Fetal Bovine Serum (FBS; 10099141, Thermo Fisher), one REGMTM SingleQuotsTM supplement pack (CC-4127, Lonza), and 1 ​mL Primocin (ant-pm-1, InvivoGen) were added to 500 ​mL REGM basal medium (CC-3191, Lonza). REGM/DMEM medium: A 1:1 mixture of REGM complete medium and DMEM complete medium was used for initial culture and expansion.

### Obtain mitochondrial fluorescence images of living cells

During this process, cells were cultured in a quadruple confocal glass-bottom culture dish, treated with the designated drugs, and subsequently stained with MitoTracker Deep Red. Live-cell imaging was then performed. The cells were placed in a small chamber within a live-cell imaging system, where the temperature and carbon dioxide concentration were maintained at 37 ​°C and 5 ​% CO_2_, respectively. Imaging was conducted using a Leica confocal microscope equipped with an oil-immersion objective at a total magnification of 1000 ​× ​. The fluorescence channel was set to Y5. Cells with moderate density and no overlap were selected to ensure accurate mitochondrial visualization. Each field of view was imaged with a z-axis thickness of 3–4 ​μm, enabling the acquisition of mitochondrial structures across multiple optical sections. Following image acquisition, preliminary denoising was performed using Thunder Analysis to minimize background noise and imaging artifacts, thereby enhancing the clarity and structural definition of mitochondrial morphology.

### Dataset size and preprocessing techniques

Our study utilized a dataset comprising 677 high-resolution (2048 ​× ​2048 pixels) fluorescence microscopy fields. To rigorously develop and validate the classification models, we employed a 5-fold cross-validation strategy. The dataset was divided into five distinct folds, for each fold, one experiments serves once as the test set while the remaining folds were used for training. The detailed distribution of fields per class (CCCP, FL3, and NC) across training and test sets for each fold is provided in Table 2.

**Table 2:** Per-fold Training/Test counts of fields for CCCP/FL3/NC.

	Training			Test		
	CCCP	FL3	Normal	CCCP	FL3	Normal
Fold 0	224	191	170	17	48	27
Fold 1	173	190	183	68	49	14
Fold 2	210	221	186	31	18	11
Fold 3	206	221	190	35	18	7
Fold 4	205	209	177	36	30	20

A multi-step preprocessing pipeline was applied to prepare the images for deep learning (Fig. S2). As significant portions of each field consist of background, we first used Mask R–CNN to extract foreground regions containing cellular structures, ensuring that subsequent analyses focused on relevant biological content. From these foreground regions, smaller image patches were extracted using a sliding window approach with a size of 112 ​× ​112 pixels and a stride of 100 pixels. To maintain data quality and prevent learning from uninformative regions, patches containing less than 50 ​% foreground content were discarded. For model input, four patches were randomly sampled from this filtered pool. This multi-patch strategy provides a robust and comprehensive representation of the mitochondrial network and reduces the risk of classification errors that could result from relying on a single, potentially uninformative patch.

### Learning image features with convolutional neural networks

Model Architecture and Training: For all classification tasks, we employed a ResNet-18 architecture pre-trained on the ImageNet dataset to leverage transfer learning. The model was trained to classify the 224 ​× ​224 pixel patches generated during preprocessing. Training was conducted over 200 epochs using the Adam optimizer with a learning rate of 1 ​× ​10^−4^ and a batch size of 128. Default PyTorch implementations were used for the learning rate scheduler and weight decay. To prevent overfitting and enhance generalization, an extensive data augmentation pipeline was applied during training, including color jittering (brightness and contrast factor of 0.8), horizontal and vertical flips, 90-degree rotations, and random resized cropping (rescaling between 50 ​% and 100 ​% of the original patch size before resizing back to 224 ​× ​224 pixels).

Classification Tasks and Inference Strategy: We trained models for three distinct classification tasks: two binary tasks (CCCP vs. NC and FL3 vs. NC) and one three-class task (CCCP vs. FL3 vs. NC). To evaluate test set performance, we implemented a robust inference strategy to ensure stable and representative predictions for each full-sized field. Rather than using a single set of patches, we adopted a random sampling ensemble approach. For each test field, four patches were randomly cropped and classified as described in Section 2.7. This process was repeated 100 times and the final classification score for the field was calculated by averaging the prediction probabilities across all 100-round Monte Carlo patch sampling per field. This approach minimizes sampling bias and provides a comprehensive assessment of mitochondrial morphology across the entire field.

## Results

### Establishment of a basic AI model for mitochondrial morphology recognition in HeLa cells

To develop an AI model for classifying mitochondrial morphologies in USCs from AD patients, we first constructed a training dataset comprising three canonical states: normal, hyperfission, and hyperfusion.

**Fig. 2:**
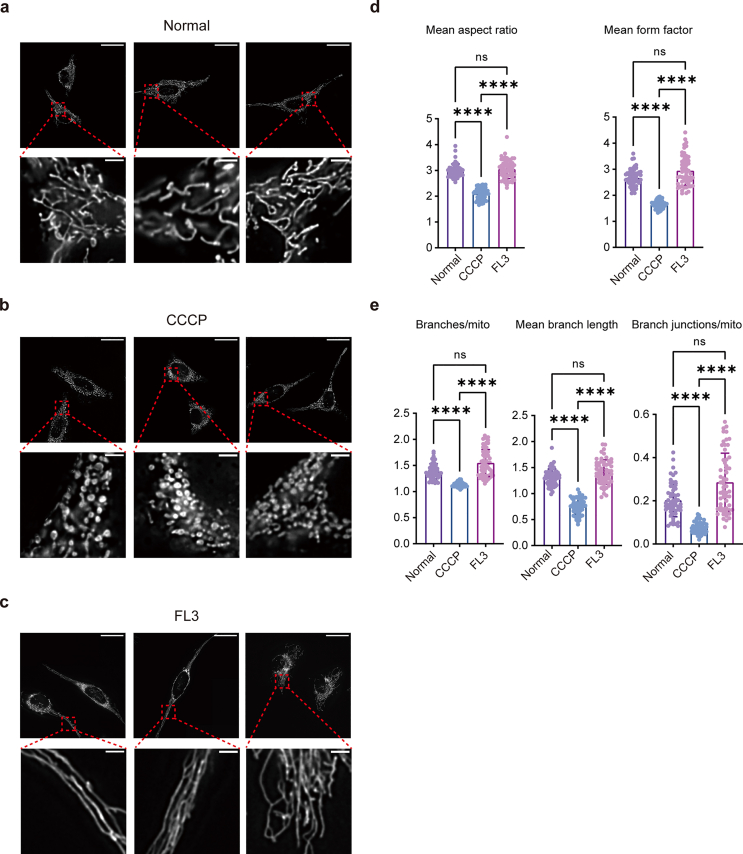
Three types of mitochondrial morphologies of HeLa cells and traditional classification. a. Representative mitochondrial morphology of normal HeLa cells (Scale bar: 40 ​μm, upper panel) and their locally enlarged image (Scale bar: 4 ​μm, lower panel). b. Representative mitochondrial morphology of 5 ​μM CCCP treated HeLa cells for 2 ​h (Scale bar: 40 ​μm, upper panel) and their locally enlarged image (Scale bar: 4 ​μm, lower panel). c. Representative mitochondrial morphology of 40 ​nM FL3 treated HeLa cells for 24 ​h (Scale bar: 40 ​μm, upper panel) and their locally enlarged image (Scale bar: 4 ​μm, lower panel). d-e. Results of *Mitochondria Analyzer* analysis of mitochondria in three states. The form factor (right) and aspect ratio (left) were used to describe the mitochondria morphology (d), the number of network branches (left), branch lengths (middle), and branch junctions (right) were used for describing the mitochondrial network (e).

To capture these baseline morphologies in living HeLa cells, we collected 197, 241, and 239 fluorescence image fields for the control, CCCP-treated, and FL3-treated groups, respectively. In normal HeLa cells, mitochondria exhibited a network-like structure, predominantly composed of curved tubular forms with a smaller proportion of punctate and elongated shapes (Fig. 2a). Hyperfission was induced using 5 ​μM CCCP for 2 ​h [47,48], resulting in fragmented, circular mitochondria and loss of network connectivity (Fig. 2b). Conversely, treatment with 40 ​nM flavagline compound 3 (FL3) for 24 ​h [49] produced elongated, interconnected mitochondria consistent with hyperfusion (Fig. 2c).

To evaluate the classification challenge, we first applied Mitochondria Analyzer, a conventional tool for morphological quantification [50]. Form factor and aspect ratio were used to describe mitochondrial shape, while network branches, branch length, and junction count quantified network complexity. Although this feature-based approach effectively distinguished CCCP-treated from control cells, it failed to reliably separate FL3-treated from control cells (Fig. 2d–e), underscoring the need for a more powerful data-driven method.

To establish a strong deep learning benchmark, we compared our proposed method against a baseline ResNet-18 model using five-fold cross-validation. Both share the same core architecture, but our model integrates key improvements—Mask R-CNN–based foreground extraction (Fig. S2), enhanced augmentation, and Monte Carlo ensemble inference for stable predictions. As summarized in Table 3, the baseline model performed well on the pronounced hyperfission phenotype (CCCP vs. NC) but was less effective at distinguishing the subtler morphological variations in the hyperfusion task (FL3 vs. NC), achieving an accuracy of only 0.8643. In contrast, our method significantly improved performance on the FL3 vs. NC classification, increasing accuracy to 0.9571. This demonstrates an enhanced capacity to capture subtle phenotypic differences. Furthermore, our model achieved near-perfect performance on the CCCP vs. NC classification across all metrics. The consistent advantage of our approach was also confirmed in a multi-class model designed to classify all three states simultaneously, underscoring its robustness and clinical applicability.

**Table 3:** Comparative performance of the proposed method against a baseline ResNet-18.

Classification Task	Method	Accuracy	Precision	Recall	F1-score	ROC-AUC
Binary: CCCP-NC	Our method	0.9951	1.0000	0.9941	0.9970	1.0000
	Baseline	0.9814	0.9963	0.9781	0.9870	0.9996
Binary: FL3-NC	Our method	0.9571	0.9950	0.9451	0.9683	0.9970
	Baseline	0.8643	0.9004	0.9039	0.8996	0.9417
3-Class: CCCP-FL3-NC	Our method	0.8826	0.9090	0.8826	0.8780	0.9765
	Baseline	0.8789	0.9016	0.8789	0.8813	0.9691

**Fig. 3:**
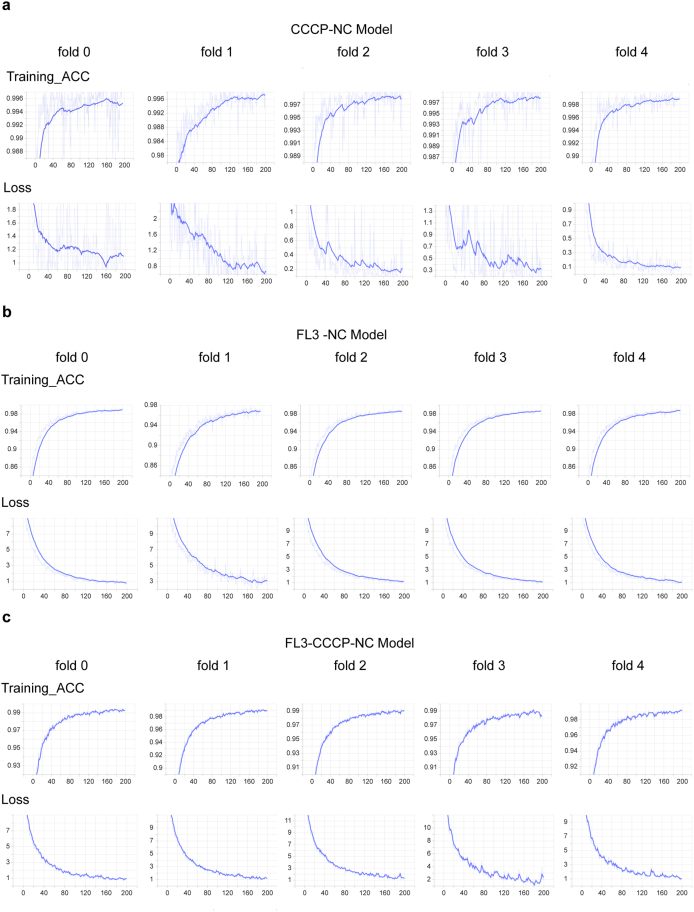
The training accuracy and loss curves for three models: CCCP-NC Model (3a), FL3-NC Model (3b), and FL3-CCCP-NC Model (3c). The curves progressively become smoother and converge with increasing training iterations. The light blue line represents the actual results, while the bright blue line denotes the smoothed curve.

Moreover, training accuracy and loss curves were plotted for three models: CCCP-NC (Fig. 3a), FL3-NC (Fig. 3b), and FL3-CCCP-NC (Fig. 3c). The curves gradually smoothed and converged over successive iterations, indicating stable and consistent training. These results suggest that the models effectively extracted relevant features from the data and achieved optimal performance. Collectively, they establish a robust foundation for mitochondrial morphology recognition and support downstream applications, including the analysis of AD-related mitochondrial phenotypes.

**Fig. 4:**
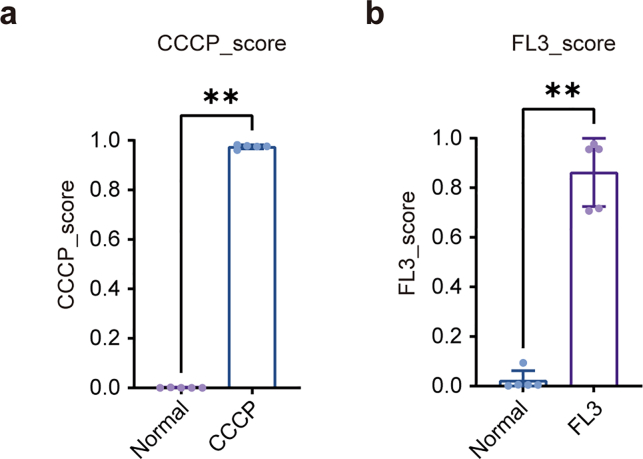
Validationof binary classification models distinguishing CCCP- or FL3-treated cells from normalcontrols. a. The scores of the validation set inthe CCCP-NC binary classification modelshows that there are significant differencesbetween the two groups. ∗∗*p<* 0.01, Errorbars represent the standard deviation (SD) (*n* = 5). b. The scores of the validation set inthe FL3-NC binary classification modelshows that there are significant differences between the two groups. ∗∗*p<* 0.01, Errorbars represent the standard deviation (SD) (*n* = 5).

Given the distinct morphological features across categories, binary classifiers were chosen to focus on category-specific characteristics while minimizing confounding effects. Unlike multi-class models, binary classifiers independently optimize decision boundaries, reducing mutual interference. Accordingly, we implemented two independent binary models: CCCP-NC and FL3-NC, generating standardized prediction scores (CCCP-score and FL3-score). Five-fold cross-validation showed that the CCCP group achieved a mean CCCP-score of 0.975 (Fig. 4a), while the FL3 group reached a mean FL3-score of 0.863 (Fig. 4b). Both models also demonstrated high accuracy in predicting normal mitochondrial images, with probabilities of 0.999 and 0.978, respectively (Table 4, Table 5).

**Table 4:** The results of five-fold cross validation for CCCP-NC Model.

Fold∖Grounp	CCCP		NC	
	CCCP_score	NC_score	CCCP_score	NC_score
Fold 0	0.9782	0.0218	0.0000	1.0000
Fold 1	0.9611	0.0389	0.0024	0.9976
Fold 2	0.9819	0.0181	0.0001	0.9999
Fold 3	0.9760	0.0240	0.0000	1.0000
Fold 4	0.9754	0.0246	0.0002	0.9998
Average	0.9745	0.0255	0.0005	0.9995

**Table 5:** The results of five-fold cross validation for FL3-NC Model.

Fold∖Grounp	FL3		NC	
	FL3_score	NC_score	FL3_score	NC_score
Fold 0	0.7172	0.2828	0.0047	0.9953
Fold 1	0.7067	0.2933	0.0940	0.9060
Fold 2	0.9760	0.0240	0.0057	0.9943
Fold 3	0.9555	0.0445	0.0013	0.9987
Fold 4	0.9565	0.0435	0.0045	0.9955
Average	0.8624	0.1376	0.0220	0.9780

### AI model accurately recognizes intermediate states of mitochondria

**Fig. 5:**
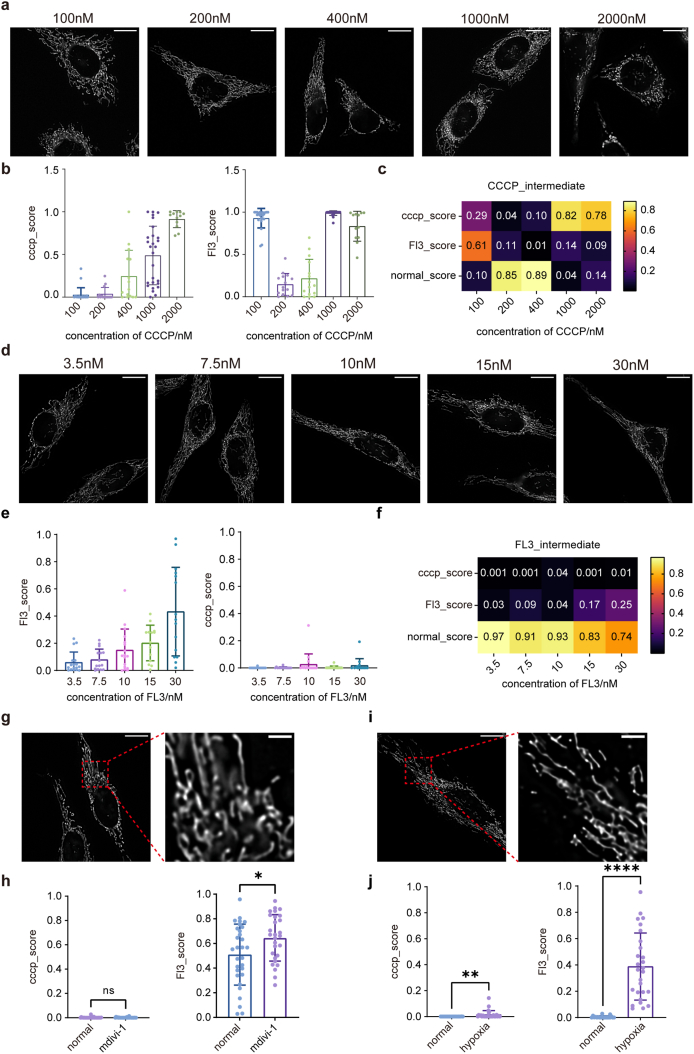
Analysis of intermediate mitochondrial morphological by AI model. a. The representative images of mitochondria in HeLa cells after treatment with 100, 200, 400, 1000, 2000 ​nM CCCP for 2 ​h. Scale bar: 20 ​μm. b. The average CCCP-scores (left) and FL3-scores (right) of CCCP group by the binary classification models. The number of microscope field corresponding to the concentration of 100, 200, 400, 1000 and 2000 ​nM is 22, 15, 15, 27 and 12, respectively. Error bars represent SD. c. The average scores of CCCP group by the FL3-CCCP-NC classification model. d. The representative images of mitochondria in HeLa cells after treatment with 3.5, 7.5, 10, 15, 30 ​nM FL3 for 24 ​h. Scale bar: 20 ​μm. e. The average FL3-scores (left) and CCCP-scores (right) of FL3 group by the binary classification models. The number of microscope field corresponding to the concentration of 3.5, 7.5, 10, 15, 30 ​nM is 17, 15, 17, 14, 15 respectively. Error bars represent SD. f. The average scores of CCCP group by the three-classification model. g. The representative images (left) of mitochondria in HeLa cells and their locally enlarged image (right) after treatment with mdivi-1 for 12 ​h. Scale bar: 20 ​μm (left), 4 ​μm (right). h. The average CCCP-scores (left) and FL3-scores (right) of mdivi-1 group by the binary classification models. The number of microscope field of normal and hypoxia is 30, 28 respectively. ∗*p* ​< ​0.05, ns, not significant. Error bars represent SD. i. The representative images (left) of mitochondria in HeLa cells and their locally enlarged image (right) after treatment with hypoxia for 3 ​h. Scale bar: 20 ​μm (left), 4 ​μm (right). j. The average CCCP-scores (left) and FL3-scors (right) of hypoxia group by the binary classification model. The number of microscope field of normal and hypoxia is 20, 27 respectively. ∗∗*p* ​< ​0.01, ∗∗∗∗*p* ​< ​0.0001. Error bars represent SD.

AI models trained solely on extreme mitochondrial morphologies often fail to capture intermediate states typical in patients’ samples, which lie between full hyperfusion and hyperfission. To address this, HeLa cells were treated with graded concentrations of CCCP (100 ​nM, 200 ​nM, 400 ​nM, 1 ​μM, and 2 ​μM) for 2 ​h to induce a continuum of mitochondrial changes (Fig. 5a). At 100–400 ​nM, mitochondria appeared largely normal, with occasional hyperfission at 400 ​nM; at 1–2 ​μM, hyperfission became prominent, including numerous ring-shaped mitochondria. Inputting these images into the binary models, the CCCP-NC model showed dose-dependent CCCP-scores, exceeding 0.5 ​at 1 ​μM and approaching 1 ​at 2 ​μM (Fig. 5b, left). Interestingly, the 100 ​nM group had a high FL3-score (mean ​= ​0.93), which decreased at 200 ​nM and then increased with higher CCCP doses (Fig. 5b, right), indicating overlapping features between early hyperfusion and hyperfission. Using the FL3-CCCP-NC model, similar trends were observed (Fig. 5c). In CCCP-treated cells, the 100 ​nM condition showed the highest FL3-score, while higher concentrations reduced FL3-scores. CCCP-scores remained low at 200 ​nM and 400 ​nM, peaking only at 1–2 ​μM.

To assess intermediate states between normal and hyperfusion, HeLa cells were treated with graded FL3 concentrations (3.5, 7.5, 10, 15, and 30 ​nM). Minimal morphological changes were observed at 3.5–7.5 ​nM, whereas mild elongation and more pronounced peripheral mitochondrial changes appeared at 10–30 ​nM (Fig. 5d). In the FL3-NC model, FL3-scores gradually increased with concentration after a slight initial decrease (Fig. 5e). CCCP-scores remained near 0 across all groups. In the FL3-CCCP-NC model, NC-scores exhibited the highest values within each group and decreased gradually as FL3-scores increased, while CCCP-scores remained low (Fig. 5f).

To validate the cross-condition generalization of the binary AI models, we treated HeLa cells with mdivi-1, a selective mitochondrial fission inhibitor [51], or exposed them to hypoxia [52,53]. Mdivi-1 treatment for 12 ​h increased FL3-scores without affecting CCCP-scores (Fig. 5g–h). Hypoxia for 3 ​h slightly elevated CCCP-scores and significantly increased FL3-scores relative to control (Fig. 5i–j).

Overall, the AI models reliably recognized intermediate mitochondrial states that are often undetectable by eye and provided quantitative scoring, demonstrating their potential to capture subtle morphological chang.

### AI model can identify USC mitochondrial morphology in AD and MCI patients

To examine whether mitochondrial morphology in urine-derived stem cells (USCs) differs among cognitively normal (CN), mild cognitive impairment (MCI), and Alzheimer’s disease (AD) individuals, we recruited three participant groups representing each cognitive state. The CN, MCI, and AD groups included 7, 10, and 9 participants, respectively, all of whom completed cognitive assessments (Table 1).

Urine samples were collected, and USCs were successfully cultured for mitochondrial fluorescence imaging. During culture, two distinct USC types were observed in samples from the same individual: spindle-shaped USCs (SS–USCs) and rice-shaped USCs (RS-USCs) [54]. Under fluorescence microscopy, the two types exhibited distinct mitochondrial morphologies. SS-USCs showed features resembling those of HeLa cells, with well-defined nuclei, larger nuclear-to-cytoplasmic ratios, and more regular mitochondrial networks. In contrast, RS-USCs displayed smaller nuclear-to-cytoplasmic ratios, less distinct nuclear boundaries, and elongated, radially distributed peripheral mitochondria (Fig. S1a).

**Fig. 6:**
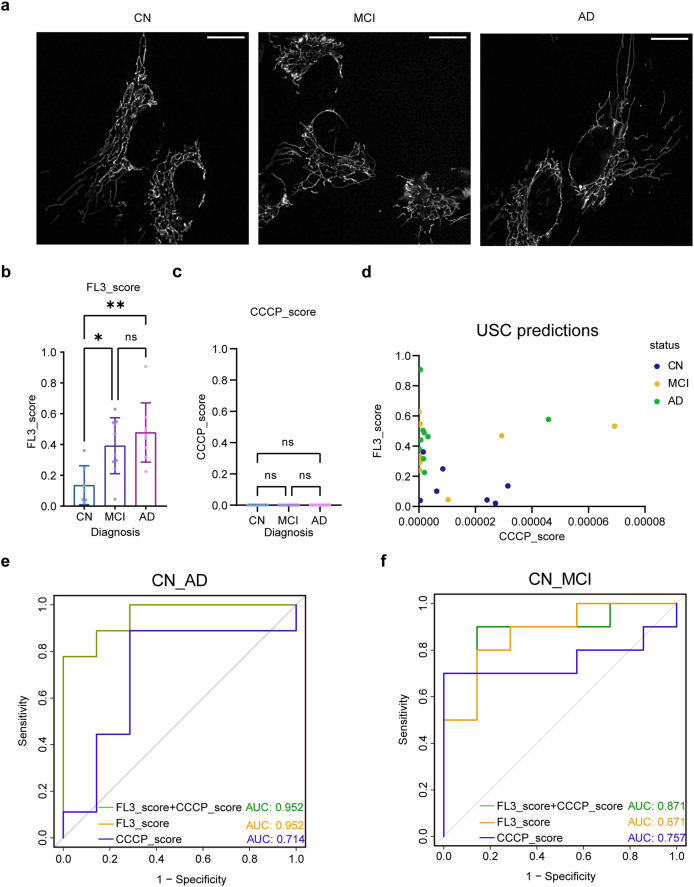
Binary AI model identify USC mitochondrial morphology in CN, MCI and AD patients. a. The representative mitochondrial images of the USCs in CN (left), MCI (middle), AD (right) participants. Scale bar: 40 ​μm. b. The average FL3-scores of USCs mitochondria in CN, MCI and AD groups. The number of patients is 7, 10, 9 participants. ∗*p* ​< ​0.05, ∗∗*p* ​< ​0.01, ns, not significant. Error bars represent SD. c. The average CCCP-scores of USCs mitochondria in CN, MCI and AD groups. The number of patients is 7, 10, 9 participants. ns, not significant. Error bars represent SD. d. The scatter plot depicting the scores for three participant groups. The horizontal axis is the CCCP-score, and the vertical axis is the FL3-score. e. ROC curves and corresponding AUC value for predicting AD by AI models. The AUC values of FL3-scores, CCCP-scores and their combination were 0.952, 0.714 and 0.952, respectively. f. ROC curves and corresponding AUC value for predicting MCI by AI models. The AUC values of FL3-score, CCCP-score and their combination were 0.871, 0.757 and 0.871, respectively.

As SS cells constituted the majority of USC populations and demonstrated more consistent morphological patterns suitable for model training, SS cell images were selected for AI-based analysis. Unless otherwise stated, subsequent references to USCs refer to SS cells. Representative mitochondrial images from CN, MCI, and AD participants are shown in Fig. 6a. Although visual differences were subtle, the AI model successfully detected group-level distinctions.

Using the FL3-NC binary model, both MCI and AD groups showed significantly elevated FL3-scores compared with CN, with no significant difference between MCI and AD (Fig. 6b). In contrast, CCCP-scores did not differ among groups (Fig. 6c). Plotting individual FL3-scores against CCCP-scores (Y-axis and X-axis, respectively) revealed clear separation between CN and cognitively impaired participants (Fig. 6d).

Consistent results were obtained with the FL3-CCCP-NC model: NC-scores declined (Fig. S1b), FL3-scores increased (Fig. S1c), and CCCP-scores remained stable with disease progression (Fig. S1d). Based on these findings, we constructed a disease prediction model using the FL3-NC classifier. The FL3-score achieved an AUC of 0.952 for AD prediction (Fig. 6e) and 0.871 for MCI prediction (Fig. 6f). Incorporating CCCP-scores did not improve performance. Overall, the FL3-score served as a robust and sensitive indicator for distinguishing cognitively normal from cognitively impaired individuals.

## Discussion

Alzheimer’s disease (AD) is now recognized as a biological continuum identifiable through biomarkers even in pre-symptomatic stages (Revised Criteria, 2024) [46]. However, current diagnostic tools such as CSF or PET are invasive, costly, and unsuitable for large-scale screening. Mitochondrial dysfunction, observed in both brain and blood during early mild MCI and AD, represents a promising biomarker target [6,13,[55], [56], [57], [58], [59], [60]]. Yet, accessible and scalable detection platforms remain unavailable. To address this gap, we developed a quantitative AI framework to analyze mitochondrial morphology in non-invasively collected urine-derived stem cells (USCs). This approach provides a convenient and repeatable platform for assessing systemic mitochondrial health. The robustness and generalizability of our models were validated through mitochondrial stress assays that generated intermediate morphological states. These AI models detected subtle mitochondrial alterations imperceptible to human observation. When applied to USCs from cognitively impaired individuals, the system identified characteristic hyperfused mitochondrial morphologies, effectively distinguishing AD and MCI patients from controls.

Our integration of AI for mitochondrial morphology analysis represents a significant advancement over traditional mitochondrial assays, which are typically labor-intensive and lack scalability. By leveraging deep learning models, we moved beyond single-organelle assessments toward a comprehensive evaluation of overall mitochondrial organization within the cellular context. The model automatically extracts and integrates features from all mitochondria in a cell, generating a unified morphological representation through convolutional layers. This holistic approach enables the robust detection of complex mitochondrial patterns that are often imperceptible to human observers, thereby enhancing sensitivity and reproducibility in morphological quantification.

The observed mitochondrial changes are consistent with previous studies reporting mitochondrial abnormalities in brain and hematopoietic tissues in MCI and AD [13,61]. Notably, we found that hyperfusion appeared as early as the MCI stage and intensified in AD, suggesting that mitochondrial remodeling reflects early disease processes. Together, these findings support the potential of AI-based USC mitochondrial profiling as a non-invasive biomarker for early screening, risk stratification, and disease monitoring.

Our strategy leverages an established computational biology paradigm: training a foundational model on a genetically stable cell line (HeLa) to learn core morphological features before validating on physiologically relevant primary cells. This approach was essential for generating the high-quality, controlled ground-truth data required to supervise the learning of extreme morphological states (hyperfusion and fission) without the confounding variability of patient-derived samples. This rationale is supported by literature; for instance, Fogo et al. demonstrated a HeLa-trained CNN could directly classify mitochondrial morphology in primary mouse neurons and brain tissue, while the Cell Painting consortium (Caicedo et al.) has established that models trained on immortalized lines (e.g., U2OS) yield broadly generalizable features [62,63]. The critical validation of our model was confirmed by its application to patient USCs, where the identification of a hyperfusion signature in MCI and AD demonstrated the translational validity of the HeLa-derived features.

Beyond methodological innovation, the biological insights gained are noteworthy. The “intermediate mitochondrial states” identified in this study are biologically meaningful rather than computational artifacts. Mitochondrial stress responses occur along a continuum—from reversible adaptation to irreversible failure—reflecting the classical dose-response relationship in cellular bioenergetics, where graded perturbations elicit proportionally scaled changes in membrane potential, metabolism, and morphology [[64], [65], [66], [67]]. To model this, we used pharmacological agents with well-characterized concentration-dependent effects. Low-dose CCCP induced mild, reversible remodeling, while higher doses caused fragmentation and apoptotic signaling [48]. Similarly, the flavagline compound FL3 produced elongation at low concentrations and cytochrome *c* release at higher levels [68]. These concentration-phenotype relationships provide a validated biological framework for defining “intermediate” mitochondrial conditions. By integrating this paradigm into our image-based classification, we link computationally derived states to mechanistically interpretable transitions, reflecting the graded nature of mitochondrial dysfunction relevant to Alzheimer’s disease.

A key strength of our approach lies in its sensitivity and objectivity. Even at low CCCP doses (200–400 ​nM), where no visible alterations were observed, the AI model detected progressive morphological deviations. Elevated FL3-scores at low CCCP concentrations suggested early hyperfusion-like responses consistent with mild stress adaptation. At higher doses, persistent overlap between hyperfusion and hyperfission signatures revealed that mitochondrial remodeling exists on a dynamic continuum. By integrating features across multiple image patches, our pipeline offers consistent, unbiased quantification that surpasses traditional, manually defined metrics. Although the CCCP-NC model could distinguish hypoxia-induced phenotypes, its low absolute scores highlight an intrinsic limitation: the model was trained primarily on extreme hyperfission, which rarely occurs under physiological conditions. Expanding the training dataset to include moderate phenotypes may further enhance clinical relevance and biological interpretability.

This study establishes a novel platform, yet several limitations highlight important directions for future research. First, although the clinical cohort size was adequate for this proof-of-concept study, expanding to larger and independent populations will be essential to validate the robustness and generalizability of the AI model’s performance. Second, as the current design is cross-sectional, it captures only a snapshot of mitochondrial alterations. Prospective longitudinal studies will be critical to determine whether these morphological features can predict the clinical progression from normal cognition to MCI and AD. Third, our current analysis treats mitochondrial morphology as an independent biomarker. Future studies should explore integrating this approach with established Alzheimer’s biomarkers—such as CSF measures, amyloid PET, or plasma p-tau—to develop a multimodal diagnostic framework and evaluate its incremental diagnostic value. Finally, improving the interpretability of the AI model will be an important goal, enabling deeper connections between the extracted image features and the underlying biological mechanisms governing mitochondrial dynamics.

Together, these considerations outline a clear trajectory for future work and highlight the translational potential of this AI-based, non-invasive mitochondrial analysis platform as an early diagnostic and monitoring tool for Alzheimer’s disease.

## Author contributions

W.Y. and W.X. conceived the idea, designed the study and directed the project. R.Y. and W.W. performed the collection of HeLa cell mitochondrial fluorescence images, patient cohort recruitment, urine sample collection, and urine-derived cell culture. W.Z. established the AI model and conducted AI-based analysis of mitochondrial images. Y.X. developed the USC culture protocol. J.W. assisted in patient cohort recruitment. R.Y. and W.W. wrote the manuscript and revised according to the comments of J.Z., Y.X., W.Y., and W.X. All authors were asked to comment on the manuscript. Y.X., J.Z., W.Y. and W.X. are the guarantors of this work and, as such, have full access to all the data in the study and takes responsibility for the integrity of the data and the accuracy of the data analysis.

## Data sharing statement

All data needed to evaluate the conclusions in the paper are present in the paper and/or the Supplementary Materials. The code for this study can be accessed via this link https://github.com/WinnieLaugh/MitoAnalysis and data will be publicly available once accepted.

## Funding

This study was supported by grants from the 10.13039/501100001809National Natural Science Foundation of China (82471394), Major Research Plan, NSFC (Grant 91954120), and Natural Science Foundation of Shanghai (NSFS, 24ZR1424900).

## Declaration of competing interest

The authors declare that they have no known competing financial interests or personal relationships that could have appeared to influence the work reported in this paper.
