# Supplementary material for: Deep learning analysis of urine-derived stem cell mitochondrial morphology as a non-invasive Alzheimer’s disease biomarker
Source: Neurotherapeutics. 2025 Dec 16;23(1):e00813. doi: 10.1016/j.neurot.2025.e00813 (PMC12976509; doi:10.1016/j.neurot.2025.e00813)
Supplement: Multimedia component 1 [file mmc1.docx]

**Deep Learning Analysis of Urine-Derived Stem Cell Mitochondrial Morphology as a Non-Invasive Alzheimer's Disease Biomarker**

Ran Yan^1#^, Wenhua Zhang^5#^, Wenjing Wang^1#^, Jiaqi Wu^4^, Jun Zhang^6*^, Yingjie Xu^2,3*^, Wei Xu^1*^, Wen Yang^2*^

Figure and Legends:


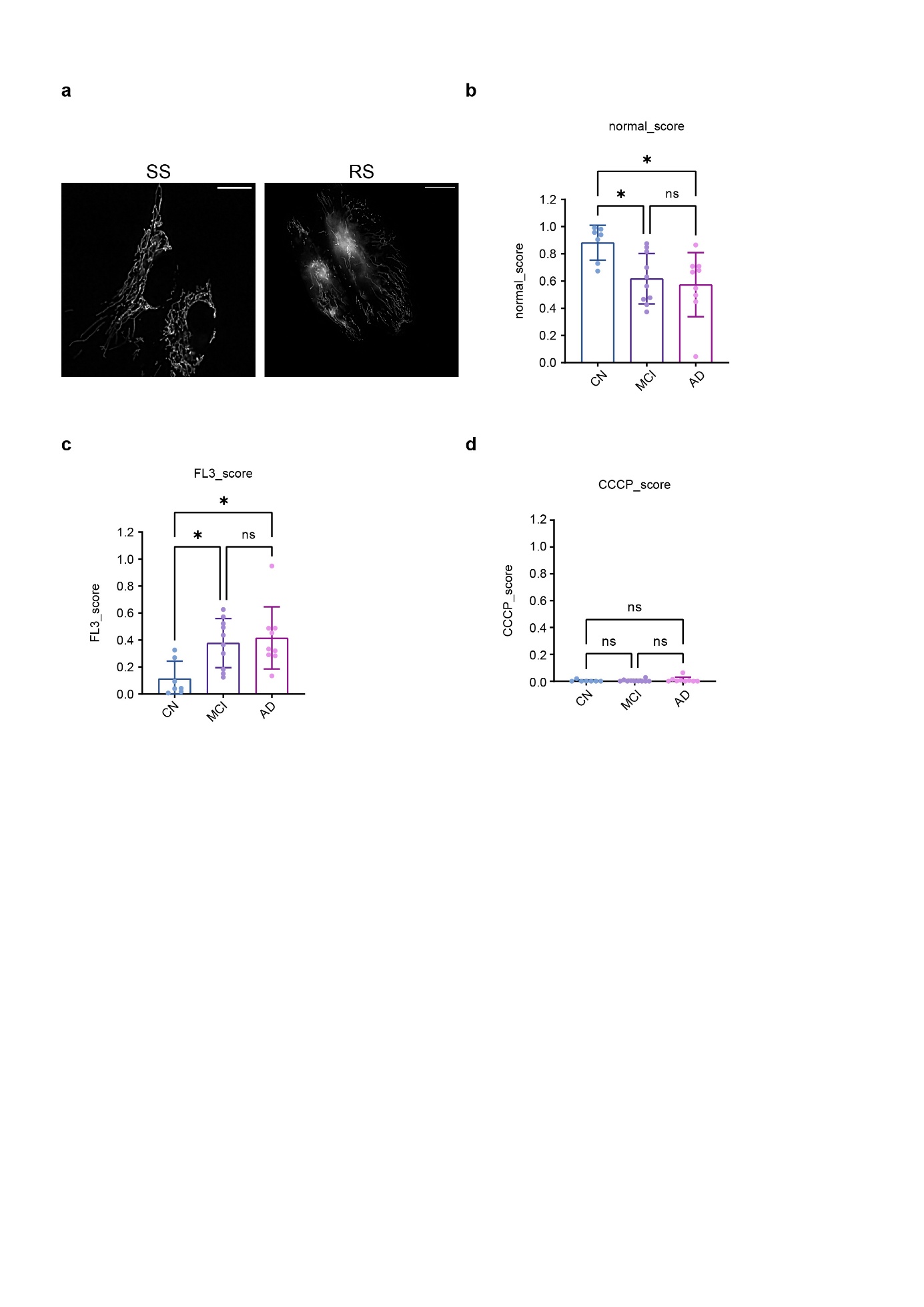


Figure S1 Three-classification AI model identifies USC mitochondrial morphology in CN, MCI and AD patients.

1. The representative mitochondrial images of the SS cells (left) and RS cells (right). Scale bar,40 μm.
2. The average NC-scores of USC mitochondria in CN, MCI and AD groups by the FL3-CCCP-NC classification model. The number of patients is 7,10, 9 participants. *p<0.05, ns, not significant. Error bars represent SD.
3. The average FL3-scores of USC mitochondria in CN, MCI and AD groups by the FL3-CCCP-NC classification model. The number of patients is 7,10, 9 participants. *p<0.05, ns, not significant. Error bars represent SD.
4. The average CCCP-scores of USC mitochondria in CN, MCI and AD groups by the FL3-CCCP-NC classification model. The number of patients is 7,10, 9 participants. ns, not significant. Error bars represent SD.


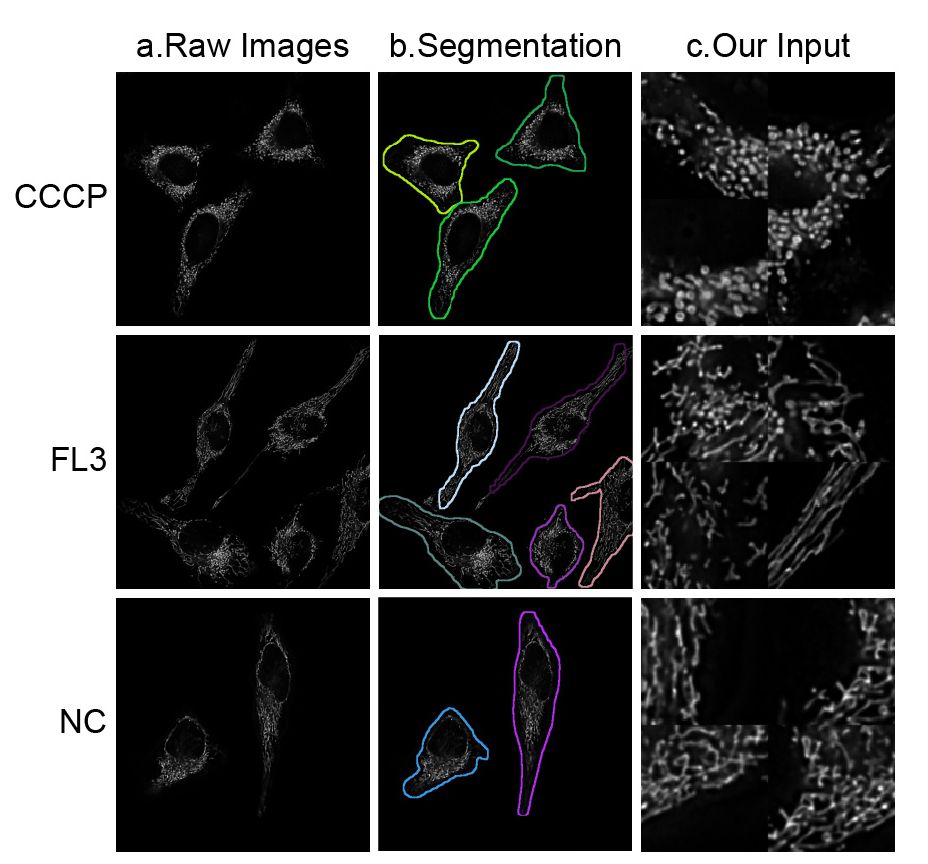
Fig. S2: Representative images for each condition (CCCP, FL3, NC) at different stages of processing.

1. Original raw fluorescence images.
2. Segmentation of individual cells using Mask R-CNN, isolating the cellular foreground from the image background.
3. A visualization of the model's four-patch input，which is composed of the segmented cellular foreground from (b) patched together. Using a group of patches ensures a robust classification by capturing a more comprehensive view of the cell's mitochondrial network and avoiding reliance on a single, potentially biased region.
